# Murine Models and Cell Lines for the Investigation of Pheochromocytoma: Applications for Future Therapies?

**DOI:** 10.1007/s12022-012-9194-y

**Published:** 2012-02-11

**Authors:** Esther Korpershoek, Karel Pacak, Lucia Martiniova

**Affiliations:** 1 Department of Pathology, Josephine Nefkens Institute, Erasmus MC-University Medical Center Rotterdam, Room Ae304, P.O. Box 2040, 3000 CA Rotterdam, The Netherlands; 2 Program in Reproductive and Adult Endocrinology, Eunice Kennedy Shriver National Institute of Child Health and Human Development, NIH, Bethesda, MD USA

**Keywords:** Pheochromocytoma, Knock-out mice, Animal models, Pheochromocytoma cell line, Imaging, Treatment

## Abstract

Pheochromocytomas (PCCs) are slow-growing neuroendocrine tumors arising from adrenal chromaffin cells. Tumors arising from extra-adrenal chromaffin cells are called paragangliomas. Metastases can occur up to approximately 60% or even more in specific subgroups of patients. There are still no well-established and clinically accepted “metastatic” markers available to determine whether a primary tumor is or will become malignant. Surgical resection is the most common treatment for non-metastatic PCCs, but no standard treatment/regimen is available for metastatic PCC. To investigate what kind of therapies are suitable for the treatment of metastatic PCC, animal models or cell lines are very useful. Over the last two decades, various mouse and rat models have been created presenting with PCC, which include models presenting tumors that are to a certain degree biochemically and/or molecularly similar to human PCC, and develop metastases. To be able to investigate which chemotherapeutic options could be useful for the treatment of metastatic PCC, cell lines such as mouse pheochromocytoma (MPC) and mouse tumor tissue (MTT) cells have been recently introduced and they both showed metastatic behavior. It appears these MPC and MTT cells are biochemically and molecularly similar to some human PCCs, are easily visualized by different imaging techniques, and respond to different therapies. These studies also indicate that some mouse models and both mouse PCC cell lines are suitable for testing new therapies for metastatic PCC.

## Introduction

Pheochromocytomas (PCC) are rare neuroendocrine tumors that arise from chromaffin cells of the adrenal medulla, or extra-adrenally (also called paragangliomas (PGL)), and they are characterized by overproduction of catecholamines, such as epinephrine and norepinephrine [1]. These tumors occur sporadically, but 30–40% occur in the context of a hereditary syndromes, most commonly multiple endocrine neoplasia type 2 (MEN 2), von Hippel-Lindau disease, neurofibromatosis type 1 (NF1), and the pheochromocytoma–paraganglioma syndrome [2]. Approximately 10% of PCC are malignant, although this frequency is much higher in patients with germline mutations in succinate dehydrogenase subunit B (*SDHB*) [3]. The management of patients with PCC follows an algorithm including biochemical testing, conventional anatomic imaging, and functional imaging. The standard treatment of benign PCCs is surgical removal of the tumor [4]. In contrast, there is no standard treatment of metastatic PCCs. While new PCC susceptibility genes have been revealed in the last decade, the pathogenesis of both benign and malignant/metastatic sporadic or genetically inherited PCCs is still not well understood.

**Fig. 1:**
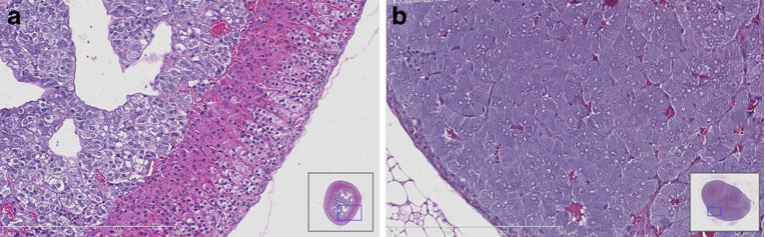
Hematoxylin eosin staining of **a** healthy mouse adrenal and **b** mouse PCC

Knock-in and knock-out mice are proven useful models to investigate the pathogenesis of human tumors including their metastatic potential. Many different mouse models that develop PCCs have been generated (Fig. 1), some of which accidentally [5–16]. An overview of all murine models is listed in Table 1.

**Table 1:** Mouse and rat models with PCC

Mouse model	AMH	PCC	PCC metastases	Other affected organs	References
Rb^+/−^		71%		Pituitary, thyroid, parathyroid, lung, pancreas	Nikitin et al. [10]
Rb^+/−^p107^−/−^		4%		Intestine, bone, lymph nodes, ovary, thyroid, lung, testis	Dannenberg et al. [6]
Rb^+/−^p130^+/−^		55%		Eye, lung	Dannenberg et al. [6]
Rb^+/−^	46%			Pituitary, lung, uterine, lymph nodes, gastro, testis, thyroid	Yamasaki et al. [16]
Rb^+/−^E2F1^+/−^	52%				
Rb^+/−^E2F1^−/−^	95%				
Rb^F2/F2-^Trp53^F2-10/F2-10^		100%			Tonks et al. [31]
p18^Ink4c−/−^	33%	8%	4% pelvic nerve	Pituitary, thyroid, testis, parathyroid, pancreas, stomach, intestine, lungs	Franklin et al. [7]
p27^Kip1−/−^	19%	24%			
p18^Ink4c−/−^p27^Kip1+/−^	42%	17%			
p18^Ink4c+/−^p27^Kip1−/−^	33%	50%			
p18^Ink4c−/−^p27^Kip1−/−^	9%	91%			
p27^kip1+/−^		Yes		Pituitary	Pellagata et al. [11]
p27^kip1^ rat		95%		Pituitary, pancreas, parathyroid, sympathetic paraganglioma (85%)	Fritz et al. [8]
p27^kip1;+/CK−^	29%			Pituitary, ovarian, lymph nodes, intestine, uterus, liver, breast, harderian	Besson et al. [36]
p27^kip1;CK−/CK−^	79%				
Pten^+/−^	10%	65%		Pituitary gland, thyroid, prostate, lung, breast	Bai et al. [5]
p18^Ink4c−/−^	29%	14%			
Pten^+/−^p18^Ink4c+/−^	6%	71%			
Pten^+/−^p18^Ink4c−/−^	11%	84%			
Pten^+/−^	24%		15% lungs	Prostate, breast, salivary gland	You et al. [22]
Ink4a^Arf+/−^Pten^+/−^	57%				
Ink4a^Arf−/−^Pten^+/−^	59%				
Pten^+/−^		23%		breast, endometrium, prostate, gastrointestin, lymphoid	Stambolic et al. [14]
Pten^loxP/loxP^	100%		35% lungs	Prostate, salivary gland	Korpershoek et al. [9]
Pten^+/−^		100%		Prostate, thyroid, intestine, endometrium, lung	Di Christofano et al. [21]
Pten^+/−^p27^Kip1−/−^		100%			
Ret^Met918Thr/Wt^	16%	2%			Smith-Hicks et al. [92]
RET^Met918Thr/Met918Thr^	100%			Hyperplasia of sympathetic ganglia	Sweetser et al. [15]
Nf1^+/−^		20%			Powers et al. [60]
Nf1^+/−^irradiated		87%			

In recent years, there has been great interest in the developing a PCC cell line that would be physiologically similar or relevant to human PCC cells and could be used for further study of genetic abnormalities, preclinical applications, testing new imaging probes and techniques, as well as the development of new radiotracers and therapeutic options. Despite some initial promising reports [17], no proven human PCC cell lines exist. In this article, we will review all mouse and rat models that develop PCC, discuss different imaging techniques, and two recently developed mouse PCC cell lines and their suitability for testing current as well as new therapies that might be used for the treatment of human metastatic PCC.

## *Pten* Knock-Out Mice

The *PTEN* gene (phosphatase and tensin homolog deleted from chromosome 10) is a tumor suppressor gene that inhibits the AKT-pathway by converting phosphatidylinositol 3,4,5 triphosphate (PIP3) into phosphatidylinositol 3,4 biphosphate (PIP2). This dephosphorylation is counteracted by PI-3 kinase (PI3K), which converts PIP2 into the active PIP3. Through PI3K, multiple pathways are triggered, many of which are associated with cell growth and survival [18]. The *PTEN* gene is one of the most frequently mutated genes in human cancer, although it has never been directly associated with human PCC [19, 20]. In contrast, several *Pten* knock-out mouse models have been reported to present with PCC at a high frequency [5, 21, 22]. Stambolic et al. found PCC in 23% (19 of 81) of conventional *Pten* ^*+/−*^ knock-out mice older than 8 months, which were, with the exception of one, all female [14]. In the study of You et al. [22], mice with identical genetic backgrounds were investigated in more detail. In addition, a combined double knock-out mouse model was created, inactivating both *Pten* and *Ink4a* ^*Arf*^. The *Ink4a* ^*Arf*^ gene encodes *p16* ^*Ink4a*^ and *p19* ^*Arf*^, which act as tumor suppressor genes and regulate the pRb and p53 pathways, respectively [23]. Four genotypes, including mono-allelic inactivation of *Pten* and mono-allelic and bi-allelic inactivation of *Ink4a* ^*Arf*^, were investigated for the occurrence of all tumor types, including the presence of PCC. Only (mono-allelic) inactivation of *Pten* led to PCC, and co-inactivation of *Ink4a* ^*Arf*^ resulted in earlier tumor presentation and its higher frequency. At a mean age of onset of 42 weeks, the *Pten* ^*+/−*^ mice showed PCC in 24%, whereas *Ink4a* ^*Arf+/−*^ *Pten* ^*+/−*^ and *Ink4a* ^*Arf−/−*^ *Pten* ^*+/−*^ displayed PCC in 57% at a mean age of 30 weeks and in 59% at mean age of 24 weeks, respectively [22]. The PCC displayed loss of parts or the entire chromosome 4, which includes a chromosomal area syntenic to human chromosome 1p, which is lost at a high frequency in human PCC [24–27]. In addition, PCC metastases were seen in lungs of approximately 15% of the *Pten* ^*+/−*^ mice. Our group has reported another conditional Pten knock-out mouse model that had PCC at a high frequency [9]. It appeared that these mice developed PCC in 30% of the mice at 7–9 months of age, 88% at 10–14 months, and 100% at 15–16 months. Furthermore, PCC metastases were found in 35% of lungs of mice at 10 months and older. This frequency of lung metastases had never been described before, indicating this model could be unique for the investigation of pathogenesis of organ metastatic PCC. In addition, the genomic alterations found in our model were different from those found in the study of You et al. [22]. The PCCs in our study displayed loss of chromosomes 6 and 19 as their main genomic alterations, whereas mouse PCCs of You et al. showed mainly (partial) loss of chromosome 4. Mouse chromosomes 6 and 19 are syntenic to human chromosomal regions that are altered in human PCC, such as chromosome 11q13, 5p15, and 22q.

Other studies combined the inactivation of *Pten* with the knock-down of other genes, such as *p18* ^*Ink4c*^ and *p27* ^*kip*^. *p18* ^*Ink4c*^ is involved in the activation of pRb, a regulator of cell division. Bai et al. generated *Pten* and *p18* ^*Ink4c*^ double knock-out (KO) mice and investigated the tumor spectrum, including adrenal tumors [5]. The homozygous *p18* ^*Ink4c*^ KO mice showed PCC at relatively low frequency (14%, 2 of 14), exclusively at 6 months and older. The Pten^+/−^ mice of 3–6 months already showed PCC in 22% (two of nine), whereas the mice of 6–15 months presented PCC in 65% (13 of 20). Heterozygous and homozygous inactivation of *p18* ^*Ink4c*^ leads to higher frequencies of tumor occurrence. The PCC penetrance was almost entirely complete (84%, 16 of 19) in *p18* ^*Ink4c−/−*^ *Pten* ^*+/−*^ mice of 6–10 months. In addition, tumors of the heterozygous and homozygous double knock-out mice were relatively larger compared with those of the *Pten* ^*+/−*^ or *p18* ^*−/−*^ mice and frequently invaded the cortex and surrounding tissues, but no metastases were reported.

## Rb Knock-Out Mice

The retinoblastoma gene family includes the *RB* gene, the *p107* gene, and the *p130* gene, which are all tumor suppressor genes. The *RB* gene is the most frequently involved gene in the pathogenesis of multiple tumors. Inactivation of the *RB* gene has been associated with familial and sporadic retinoblastomas, small cell lung carcinomas, and osteosarcomas [28, 29]. In addition, inactivation of Rb has also been associated with chromosomal instability, cancer progression, and activation of angiogenesis [30]. RB functions as an essential regulator of cell cycle progression. Several research groups have created Rb knock-out mice, often with heterozygous or homozygous inactivation of an additional gene [6, 10, 16, 31].

One study investigated heterozygous inactivation of *Rb* in a conventional knock-out mouse model and demonstrated PCC in 71% of the mice (22 of 31 mice), of which 14% showed bilateral adrenal medulla hyperplasia in approximately half of the mice [10]. Other tumors that occurred at a high frequency in these mice were pituitary tumors (100%), medullary thyroid carcinoma (95%), and corresponding lung metastases (68%).

Another study combined heterozygous inactivation of *Rb* with homozygous knock-down of *p130* and *p107* [6]. These enzymes, including RB, can repress transcription from E2F-responsive promoters and are regulated by cell–cycle-dependent phosphorylation [32, 33], but act on different E2Fs [34]. The study of Dannenberg et al. showed PCC in 55% of the *Rb* ^*+/−*^ *p107* ^*−/−*^ mice (6 of 11 mice) and in 4% of the *Rb* ^*+/−*^ *p130* ^*−/−*^ (2 of 53 mice) [6]. Besides the different penetrance of PCC in these models, there was also a difference in occurrence of other tumors.

A third study investigated an *Rb* ^*+/−*^ knock-out model in combination with heterozygous or homozygous inactivation of *E2f1* [16]. Adrenal medullary hyperplasia was reported in 46% of the *Rb* ^*+/−*^ mice, in 52% of the *Rb* ^*+/−*^ *E2f1* ^*+/−*^ mice, and in 95% of the *Rb* ^*+/−*^ *E2f1* ^*−/−*^ . In contrast to the increasing frequency of PCC occurrence in the *Rb* ^*+/−*^ *E2f1* ^*−/−*^, pituitary adenocarcinomas occurred less frequently in these mice (62%) compared with a nearly full penetrance of the *Rb* ^*+/−*^ and *Rb* ^*+/−*^ *E2f1* ^*+/−*^ mice.

Recently, another study published a conditional knock-out mouse model that used the Cre-lox system to inactivate the *Rb* and *Trp53* gene [31]. Inactivation was accomplished by removal of *Rb* exon 2 and *Trp53* exons 2 to 10 by Cre recombinase. Cre recombinase expression was driven from the *TEC1* transgene using elements of *tyrosine* transcriptional elements, which are active in the development of a subset of neural crest-derived tissues including the adrenal medulla. All of the *Rb* ^*F2/F2*^ */Trp53* ^*F2-10/F2-10*^ */TEC1* ^*+/−*^ mice (*n* = 13) showed bilateral PCCs. Catecholamine synthesis was relatively intact, but storage of catecholamines was altered as shown by the heterogeneous and lower levels of immunohistochemical staining for chromogranin A and synaptophysin, and the empty and much smaller vesicles seen by electron microscopy of the PCC [31]. In addition to PCC, one of these mice showed a tumor in the neck region, suggested to be a distant metastasis by the authors. However, this could also have been a paraganglioma, as the carotid body is known to produce tyrosine in rats [35].

## *p18*^*INK4C*^ and *p27*^*Kip1*^ Knock-Out Mice and Rats

*p18* ^*INK4C*^ and *p27* ^*Kip1*^ belong to the family of cyclin-dependent kinase (CDK) inhibitors, which are classified into two families: The CIP/KIP family members are known to inhibit a variety of cyclin–CDK complexes, whereas INK4 family members specifically inhibit CDK4/CDK6 [36, 37]. All CDK inhibitors are involved in regulation of the cell cycle and result in G1 arrest.

*p18* ^*INK4C*^ has been described as a tumor suppressor gene in human glioblastomas [38], and *p18* ^*INK4C*^ mutations have also been demonstrated in *RET*-mutated PCC [39]. *p18* ^*INK4C*^ knock-out mouse models present with organomegaly and a disproportionately enlarged pituitary gland, spleen, thymus, and adrenal gland [40]. Pheochromocytomas occurred in 8.3% of the *p18* ^*−/−*^ mice (2 of 24 mice aged 8 months or older), 23.8% of the *p27* ^*−/−*^ mice (5 of 21), 17% of the *p18* ^*−/−*^ *p27* ^*+/−*^ mice (4 of 24), 50% of the *p18* ^*+/−*^ *p27* ^*−/−*^ mice (three of six), and in 91.3% of the *p18* ^*−/−*^ *p27* ^*−/−*^ double knock-out mice (21 of 23) [7]. The study also combined *p18* knock-out mice with *p21* knock-out mice, and both p21^-/−^ as well as p18^−/−^p21^−/−^ did not display PCC. Of the latter group, one mouse developed a PCC metastasis at the pelvic nerve.

Mouse and rat models with inactivated or mutated *p27* ^*Kip1*^ also presented with a multiple endocrine neoplasia-like syndrome (MEN 4). Both the *p27* ^*Kip1*−/−^ knock-out mice and the *p27* ^*Kip1*^ mutated rats developed pheochromocytomas, the latter of which was in 95% of cases [8, 11]. In addition, these rats also developed sympathetic PGLs [8]. These tumors were investigated for genomic imbalances and approximately 30% displayed loss of chromosomes 8 and 19 [13]. The chromosomal region 8q31–q32, showing the highest frequency of loss (29%), is syntenic to human chromosome 3q21.3–q24, which is lost frequently in MEN2-related as well as sporadic PCC [13, 27, 41]. Loss of chromosome 19p12–p14 occurred in 30% of the rat PCC and is syntenic to human chromosome 16q21. This chromosomal region has not been reported to be altered in human PCC or PGL but has been associated with other tumors such as retinoblastoma and papillary thyroid carcinoma [13, 42–44]. Furthermore, gene expression profiling was performed on these MEN-associated rat PCC, which revealed a neural precursor cell-like signature [45] similar to the expression profile of PCC of the *NF1* knock-out mice described by Powers et al. [46] Recently, germline mutations in the *p27* ^*KIP1*^ gene have been associated with a novel type of multiple endocrine neoplasia syndrome type 4 in humans (reviewed extensively by Maroni and Pellegata) [47] but was also found in another study [48].

## *RET*^*MEN2B*^ transgenic mice

RET (rearranged during transfection) is a receptor tyrosine kinase, located on chromosome 10q11.2, and is a proto-oncogene. RET is a receptor for the glial cell line-derived neurotrophic factor family, which plays a role in a number of biological processes such as cell survival, differentiation, and migration [49]. Mutations in *RET* cause the multiple neuroendocrine neoplasia type 2 (MEN 2) syndrome, which is subdivided into MEN 2A and MEN 2B [50]. These subtypes are clinically different, but both present with PCC and medullary thyroid carcinomas. Studies have revealed that activation by RET-mutant proteins result in activation of the RAS/RAF/MAPK, and PI3K/AKT pathways [51]. A *RET* ^*MEN2B*^ transgenic mouse model that has been created to investigate the pathogenic effect of a MEN2B-specific mutation, also developed PCC [15]. No specific percentage of PCC penetrance was mentioned in the report, but besides adrenal tumors, the mice also developed benign neuroglial tumors in the entire sympathetic nervous system, which seem to be histologically identical to human ganglioneuromas [15].

## Nf1 Knock-Out Mice

*NF1* (neurofibromin gene) is a tumor suppressor gene located on chromosome 17q11.2 and inhibits the RAS/ERK and AKT pathway by dephosphorylating active RAS to an inactive form [52]. Germline mutations cause neurofibromatosis type 1, which is characterized by café-au-lait spots, Lisch nodules in the eye, fibromatous tumors of the skin, and PCCs at a low frequency (0.1–5.7%) [53]. A heterozygous *Nf1* knock-out mouse model was created, which carried a heterozygous germline mutation in exon 31 that was representative for a mutation found in human NF1 patients [54]. In total, 250 *Nf1* ^*+/n31*^ mice were investigated for the typical clinical characteristics that occur in human NF1 patients, but no neurofibromas or pigment defects were detected. Forty of these mice were studied more closely and revealed PCC in approximately 15% of the mice, which all displayed loss of the wild-type allele, and stained positive for phenylethanolamine *N*-methyltransferase, so were able to produce epinephrine [54, 55]. Four cell lines were cultured from the Nf1^+/n31^ mouse PCC, which displayed loss of chromosomes 4 and 9, which are homologous to chromosomal regions that show loss in human PCC. Mouse chromosome 4 is syntenic to a part of chromosome 1p, and mouse chromosome 9 is syntenic to regions of human chromosomes 3p, 3q, and 11q. The cell lines will be described in more detail below.

## Sdhb/d Knock-Out Mice

The *SDHB* and *SDHD* genes encode two subunits of mitochondrial complex II, and inactivating mutations in both genes have been associated with PCCs and PGLs in humans [56]. One study investigated the Sdh activity of heart samples in an *Sdhb* KO mouse model, heterozygous for a deleterious mutation in the Sdhb gene (exon 2 deletion), and found a 40% decreased activity compared with the heart samples of healthy animals. The presence of PCC or PGLs was not reported in this paper. Another study generated a conventional knockdown of *Sdhd* in a mouse, by removal of the entire third exon, to create a mouse model for PCCs and PGLs [57]. In addition, these mice were crossed with *H19* knock-out mice, which is postulated as a modifier gene of *Sdhd* tumorigenesis, to investigate if inactivation of these genes would lead to initiation or enhancement of tumor development. Inactivation of both *SDHD* alleles resulted in embryonic lethality and of one allele in a healthy phenotype without evidence of PCC or PGL.

## Mouse PCC-Related Genes in a Common Pathway and Their Relation with Human PCC

**Fig. 2:**
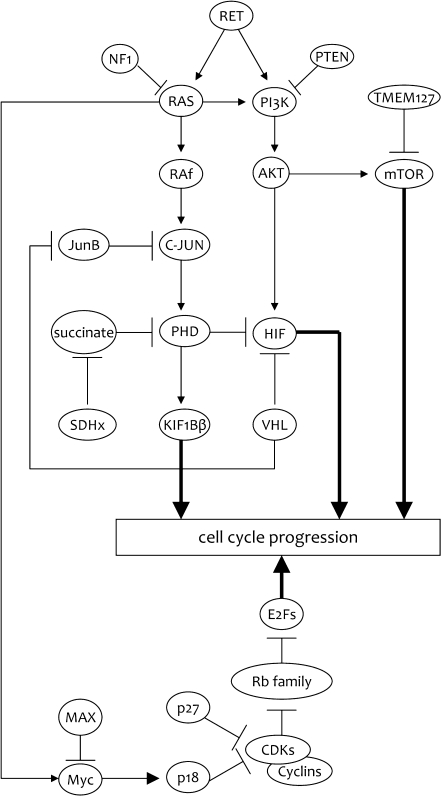
A proposed common pathway for genes that are associated with the pathogenesis mouse and/or human PCC [34, 36, 37, 51, 52, 60–62, 95–101]

If we investigate the genes involved in human and mouse PCC in more detail and focus on how they are related, a common pathway could be proposed (Fig. 2). Most of the mouse PCC-related genes are involved in the regulation of the G1 to S phase of the cell cycle, including CDK inhibitors. All CDK inhibitors result in G1 arrest when overexpressed in transfection. Two of these cell cycle-related genes, *p18* ^*INK4C*^ and *p27* ^*KIP1*^, have also been associated with human PCC. Somatic *p18* ^*INK4C*^ gene mutations co-occurred with somatic and germline *RET* mutated PCC and medullary thyroid cancer [39]. Furthermore, *p27* ^*KIP1*^ mutations were associated with the MEN type 4 syndrome, but not with PCC [11, 48, 58]. Downregulation of *p18* ^*INK4c*^ and *p27* ^*KIP1*^ expression appears an essential step in the tumorigenesis of *RET*-related tumors, and both genes are regulated by RET, of which downregulation of p18^INK4C^ is through *N*-Myc [59, 60]. *PTEN* mutations have never been associated with PCC in humans, but PTEN is an important key player in regulating the AKT and mammalian target of rapamycin (mTOR) pathways, which are altered in human PCC. In addition, mutations in patients with PCCs have recently been described in *TMEM127*, which is a negative regulator of the mTOR pathway [61] and *MAX*, the MYC-associated factor X gene, which is a tumor suppressor [62].

## An Animal Model Derived from Nf1 Mouse PCC Cells

**Fig. 3:**
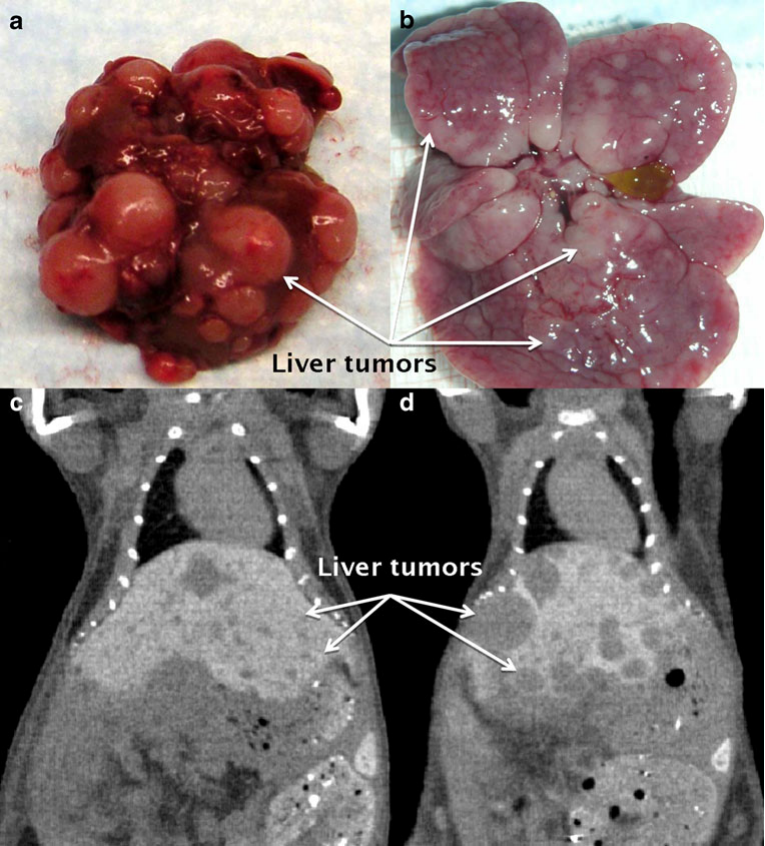
**a** Liver pathology after tail vein injection of 10 million MPC cells. 7 weeks post injection; PCC tumors almost replaced liver parenchyma that usually causes death of mice. **b** 2 weeks post injection of MPC cells, highly mitotic liver metastases were harvested and cultured to create MTT cell line. **c**, **d** A comparison of tumor growth rate 4 weeks post-injection between MPC and MTT cells using microCT. **c** One million of MPC cells, **d** one million of MTT cells injected tail vein. The aggressive growth rate of liver tumors after MTT cell injection is significant. While MPC-derived liver tumors reached approximately 0.7 mm in diameter, the MTT-derived tumors reached 2–8 mm in diameter

PCC is a rare condition, with limited availability of tumor tissue. The existence of a PCC cell line would provide us with unlimited tumor cells for the investigation of all kinds of therapies. Unfortunately, no human PCC cell line exists, but there are cell lines from mouse and rat PCC. The rat PC12 cell line has already been reviewed extensively, so we will focus on the mouse pheochromocytoma (MPC) cell line created from a *Nf1* KO mouse PCC by the group of Tischler et al. [63]. These MPC cells were the basis for the mouse tumor tissue (MTT) cells that will also be discussed below. The main advantages of the MPC cell line include the genetic and biochemical resemblances to human PCCs, such as the expression of substantial levels of the epinephrine-synthesizing enzyme phenylethanolamine *N*-methyltransferase, and expression of high levels of the receptor tyrosine kinase, Ret [64, 65]. These biochemical features are characteristics of sporadic and familial human PCCs but not of the rat PC12 cells [64–66]. MPC cells were originally used to create a subcutaneous mouse model [67]. Subsequently, MPC cells were injected via the tail vein in nude female mice, which resulted in a model of metastatic PCC, showing numerous liver lesions in more than 90% of the injected animals. Liver lesions were detectable as early as 4 weeks after injection of MPC cells, using very sensitive imaging techniques (Fig. 3). Non-hepatic PCC lesions developed usually in the fifth week or later and included the adrenal glands (30%), which were often bilateral, ovaries (30%), lungs (20%), kidneys (10%), bones (spine and hip bone area) (10%), and, much less frequently, muscles [68].

The metastatic PCC model provides a unique platform for studying physiologic changes and interactions, especially since implants can be created in all major organs. Gene expression microarray analysis can be used to screen tumors for differentially expressed genes between two or more groups. Both animal models (metastatic and subcutaneous) revealed different gene expression of metastasis-associated genes. Using quantitative real-time PCR, expression of five genes (Metap2, Reck, S100a4, Timp2, and Timp3) were verified as significantly lower in liver than in subcutaneous tumors. Downregulation of these genes has been previously associated with malignancy of PCCs [67]. Subcutaneous tumors are simple to monitor but do not resemble the microenvironment of animal/human body. Therefore, a metastatic model is more relevant in the study of tumor biology, evaluation of response to treatment, and for monitoring toxicity after drug administration.

## Bench-to-Bedside Approach

Since conventional management of clinical PCC includes biochemical testing and anatomical and functional imaging modalities, the same approach should be used in metastatic animal models. As a miniaturized version of the traditional clinical computed tomographic (CT) imaging system, small-animal microCT scanners are capable of providing tumor localization and tumor growth monitoring with excellent spatial resolution. However, this imaging technique requires the use of specific contrast agents for proper localization of soft tissue tumors. The hepatobiliary contrast agent Fenestra™Liver Contrast (Fenestra™LC, Advanced Research Technologies, Inc.) is ideal for liver tumors localization, and its utility was described in detail previously [68–70]. Other anatomical imaging techniques, such as magnetic resonance imaging (MRI), provide certain advantages over microCT. T2-weighted MRI, for instance, does not require contrast agents and is rapid to perform, with whole body images obtained in less than 5 min [68]. However, none of the anatomical imaging techniques properly access tumor physiology.

PCCs are characterized by the presence of membrane norepinephrine transporters (NET), vesicular monoamine transporter (VMAT) system, catecholamine synthesis and storage mechanisms, and amino acid transporters, all of these providing a potential molecular imaging target. The dopamine analogue [^18^ F]-dopamine ([^18^ F]-DA) [71, 72] enters cells via the membrane NET [73, 74] and is then translocated via the VMAT into storage vesicles, where the radioactivity is concentrated. [^123^I]-MIBG uses similar mechanisms to enter these cells, but importantly, its affinity to NET is lower compare with [^18^ F]-DA. Uptake in these tumors depends on both NET and VMAT and subsequently storage in vesicles. Therefore, if PCC metastases do not express NET, other (less specific) radiotracers can be used for detection and localization of tumors, including [^18^ F]-DOPA. [^18^ F]-DOPA is an analog of l-3,4-dihydroxyphenylalanine (l-DOPA) [75], a precursor of catecholamines (dopamine, norepinephrine, and epinephrine). These two radiotracers revealed great discrepancy in uptake in mouse subcutaneous and metastatic PCCs. A recently published study [76] demonstrated that the same MPC cells, injected intravenously and subcutaneously, create physiologically different tumors, as could be expected, in relation to the microenvironment of the tumor cells. While [^18^ F]-DA and [^18^ F]-DOPA PET performed equally well for the detection of ovarian metastatic tumors, [^18^ F]-DOPA PET showed superiority to [^18^ F]-DA PET in the detection of hepatic, lung, and subcutaneous tumors. In particular, subcutaneous tumors were detected only with [^18^ F]-DOPA PET. Comparisons of the in vitro uptake of both radiotracers by MPC cells confirmed the importance of the NET for uptake of [^18^ F]-DA and also confirmed that VMAT2, and to a lesser extent VMAT1, is important for its retention in catecholamine storage vesicles. The differences in uptake of these two radiotracers may be relevant to recent clinical findings of differences in functional imaging characteristics of various PCC, depending on their underlying mutations and nature [77]. For example, in patients with malignant paragangliomas due to succinate dehydrogenase subunit B mutations, [^18^ F]-DA is superior to [^18^ F]-DOPA for localization of metastases (reverse to the present animal model of PCC), whereas in other patients with so-called head-and-neck paragangliomas, [^18^ F]-DOPA is superior to [^18^ F]-DA [78].

## More Aggressive Mouse Model of Metastatic PCC

Recently, another model of metastatic PCC has been developed, following the approach revealed from gene expression differences between subcutaneous and liver metastases [79]. Liver lesions behaved more aggressively compared with the rest of the non-hepatic PCC metastases, and thus, disaggregated tumor cells from hepatic metastases were isolated and cultured, resulting in a more aggressive sub-line named MTT cells (Fig. 3a, b). The MTT cells, when injected via the tail vein, produce metastatic disease that retains the histological and biochemical features of PCC, while at the same time representing a closer convergence with the aggressiveness seen in the human disease. Martiniova et al. confirmed that the MTT cells derived from MPC maintain a PCC phenotype by measuring intracellular catecholamines; the expression of TH by immunocytochemistry; TH and PNMT gene expression by quantitative real-time polymerase chain reaction (qRT-PCR); and the morphologic presence of dense-core secretory granules by electron microscopy, a diagnostic feature of PCC [79]. Elevated norepinephrine levels were also detected in MTT-derived liver tumors compared with normal liver tissue. Monitoring of tumor growth by microCT/MRI in the MTT model clearly demonstrated its practicality, in particular, for pre-clinical testing of novel treatment, because of the greater number of lesions achieved and shorter development time (Fig. 3c, d).

Since more aggressive MTT cells could again show close similarities to human metastatic PCC, the newly generated MTT and MPC microarray data were mined against a previously generated human PCC microarray database [80]. Genes that were twofold up- or downregulated were accounted for and compared with genes in benign vs. malignant human microarray. The 47 genes were put in the ingenuity pathway analysis to determine if these genes are part of any biological pathway. As a result, seven genes were found to be part of a network. qRT-PCR was performed on the mouse MPC and MTT cells and also on a different set of human metastatic and non-metastatic PCCs to compare the in vitro/animal data to the human samples. This validation revealed two genes (FRK, *P* = 0.0027, and KRT8, *P* = 0.0003) that were significantly downregulated in MTT cells compared with MPC confirming the microarray analysis [79].

## Application of PCC Metastatic Models in Preclinical Treatment Assessment

Here, we will introduce three different approaches, how a model of metastatic PCC was used in preclinical treatment assessment. All these approaches are ready for translation into the clinic in the near future. In the process of experimental drug screening, all were evaluated in vitro using MPC or MTT cells. For the purpose of assessment of targeted therapy, the expression of transporters and proteins was evaluated in both human and mouse metastases. When a candidate drug was selected, the appropriate imaging technique to monitor tumor response longitudinally was chosen.

### Utilization of Increased Expression of Interleukin (IL)-13 IL-13Rα2 in PCCs

Gene expression microarray analysis has been used to screen for genes and signaling pathways that play pivotal roles in cellular transformation, tumor progression, and development of metastases. Increased expression of interleukin (IL)-13 IL-13Rα2 in PCC was identified through human and mouse microarray analysis as a potential target for directed therapy with an immunotoxin consisting of IL-13 and truncated *Pseudomonas* exotoxin A (IL-13PE). The IL-13Rα2 binds IL-13 with high affinity and is over-expressed in a variety of cancers [81, 82]. Experimental drug testing started on both subcutaneous and metastatic tumors, with intratumoral injection of 100 μg/kg IL-13PE or PBS for three consecutive days. IL-13Rα2 expression was confirmed in both subcutaneous and liver tumors, as well as in human PCC by quantitative RT-PCR [83]. Conversely, normal adrenal medulla tissue did not reveal expression of IL-13Rα2. IL-13PE treatment resulted in significant suppression of subcutaneous tumors compared with placebo after 3 days initial treatment (*P* = 0.0021), but not of liver tumors, which required intratumoral delivery of IL-13PE [83]. This treatment could still be used, due to the high specificity for most of human PCCs with all gene mutations, however, in limited number of cases. Drug would have to be administered using guided CT or MRI intratumoral injections.

### The Utilization of Histone Deacetylase Inhibitor Romidepsin Evaluated by [^18^ F]-DA PET

The advantage of positive results from [^18^ F]-DA functional imaging and identifying of NET expression in metastases in the metastatic mouse model allowed utilizing this animal model in experimental treatment through the modification of NET expression in PCC. One of the most effective treatments for malignant PCC includes [^131^I]-MIBG [84–90] that specifically targets chromaffin and PCC cells via the NET [88, 89, 91]. Unfortunately, only 30% of patients show a tumor response to [^131^I]-MIBG [86]. This disappointing response rate is most likely related to the under-expression of NET and low number of storage granules that results in lower [^131^I]-MIBG concentrations within the tumor cells. Thus, an increase of NET and of the number of storage granules might improve the response rate of such treatment. Liver tumors in MPC mouse model revealed a wide range of NET expression as well as [^123^I]-MIBG/[^18^ F]-DA uptake [92]. Testing two structurally different HDACi, romidepsin and trichostatin A, in MPC cells in vitro and in a mouse model of metastatic PCC in vivo resulted in modification of NET transporter expression in liver metastases. The following clinical approach was used in this study: Mouse/patient was initially scanned with [^123^I]-MIBG/[^18^ F]-DA. In general, those positive on [^123^I]-MIBG scintigraphy are good candidates for [^131^I]-MIBG treatment, but there is a need to treat also those patients with negative [^123^I]-MIBG scintigraphy. After series of tests, a pretreatment with a single dose of romidepsin, 2.5 mg/kg, was used to evaluate its effect on isotope accumulation in the tumors. In conclusion, treatment with the HDAC inhibitors romidepsin and trichostatin A increased [^123^I]-MIBG and [^18^ F]-DA uptake in MPC cells in vitro and in vivo in liver metastatic lesions, through the upregulation of the cell membrane NET [92]. These data support the notion that this approach may be used clinically to augment the therapeutic efficacy of [^131^I]-MIBG in patients with advanced malignant PCC, paraganglioma, and other related tumors such as neuroblastoma.

### A Complex Drug Evaluation and Treatment of Liver Metastases Using a Small Molecule Inhibitor of Serine/Threonine Protein Phosphatase 2A in Combination with Conventional Chemotherapy

The failure of cytotoxic cancer regimens to cure the most drug-resistant, well-differentiated solid tumors has been attributed to the heterogeneity of cell types that differ in their capacity to grow, differentiate, and metastasize. Data also supports that MPC cells and PCC metastasis are in different cell cycle phases, and thus any experimental treatment approach would have only short-term responses. A new approach of investigating the effect of LB1, a small molecule inhibitor of serine/threonine protein phosphatase 2A (PP2A), was proposed and presented by Lu et al. [93], in a neuroblastoma xenograft model, where, by modifying the tumors’ cell cycle, they increased chemotherapeutic (temozolomide) effectiveness. For treatment of liver metastases, LB1 was continuously released by surgically inserting a small osmotic pump into the peritoneal cavity in mice. Longitudinal MRI was used to monitor lesions from MPC injection until the end of the treatment. The effect of LB1 and temozolomide, a standard chemotherapeutic agent that alone only transiently suppressed the growth and regression of metastatic PCC, was evaluated. This new approach resulted in long-term, disease-free survival of up to 40% of animals bearing multiple intrahepatic metastases, a disease state that the majority of patients die from. Inhibition of PP2A was associated with prevention of G1/S phase arrest by p53 and of mitotic arrest mediated by polo-like kinase 1 (Plk-1) [94]. The elimination of DNA damage-induced defense mechanisms, through transient pharmacologic inhibition of PP2A, is proposed as a new approach for enhancing the efficacy of non-specific cancer chemotherapy regimens against a broad spectrum of low growth fraction tumors very commonly resistant to cytotoxic drugs.

## Conclusion

PCC of several animal models have been shown to resemble human PCC. Most mouse and rat models presented with benign PCC, but two studies reported lung metastases. Because no human PCC cell line exists, other cell lines would be very relevant to determine what chemotherapeutics could be used for the treatment of metastatic PCC. It would be beneficial to create cell lines of the “spontaneously” metastasizing PCC of *Pten* knock-out mice, but this has not been successful yet. As an alternative, the MPC and MTT cell lines could be used, which have been created from radiated *Nf* knock-out mouse PCC. Both the spontaneous metastatic PCC mouse models and mice injected with MPC and MTT cell lines might be useful for the investigation of currently available targeted cancer therapies.

## References

[1] Eisenhofer G, Pacak K, Huynh TT (2010). Catecholamine metabolomic and secretory phenotypes in phaeochromocytoma. Endocr Relat Cancer.

[2] Lenders JW, Eisenhofer G, Mannelli M, Pacak K (2005). Phaeochromocytoma. Lancet.

[3] Gimenez-Roqueplo AP, Favier J, Rustin P (2003). Mutations in the SDHB gene are associated with extra-adrenal and/or malignant phaeochromocytomas. Cancer Res.

[4] Petri BJ, van Eijck CH, de Herder WW, Wagner A, de Krijger RR (2009). Phaeochromocytomas and sympathetic paragangliomas. Br J Surg.

[5] Bai F, Pei XH, Pandolfi PP, Xiong Y (2006). p18 Ink4c and Pten constrain a positive regulatory loop between cell growth and cell cycle control. Mol Cell Biol.

[6] Dannenberg JH, Schuijff L, Dekker M, van der Valk M, te Riele H (2004). Tissue-specific tumor suppressor activity of retinoblastoma gene homologs p107 and p130. Genes Dev.

[7] Franklin DS, Godfrey VL, O’Brien DA, Deng C, Xiong Y (2000). Functional collaboration between different cyclin-dependent kinase inhibitors suppresses tumor growth with distinct tissue specificity. Mol Cell Biol.

[8] Fritz A, Walch A, Piotrowska K (2002). Recessive transmission of a multiple endocrine neoplasia syndrome in the rat. Cancer Res.

[9] Korpershoek E, Loonen AJ, Corvers S (2009). Conditional Pten knock-out mice: a model for metastatic phaeochromocytoma. J Pathol.

[10] Nikitin AY, Juarez-Perez MI, Li S, Huang L, Lee WH (1999). RB-mediated suppression of spontaneous multiple neuroendocrine neoplasia and lung metastases in Rb+/- mice. Proc Natl Acad Sci U S A.

[11] Pellegata NS, Quintanilla-Martinez L, Siggelkow H (2006). Germ-line mutations in p27Kip1 cause a multiple endocrine neoplasia syndrome in rats and humans. Proc Natl Acad Sci U S A.

[12] Powers JF, Tischler AS, Mohammed M, Naeem R (2005). Microarray-based comparative genomic hybridization of pheochromocytoma cell lines from neurofibromatosis knockout mice reveals genetic alterations similar to those in human pheochromocytomas. Cancer Genet Cytogenet.

[13] Shyla A, Holzlwimmer G, Calzada-Wack J (2010). Allelic loss of chromosomes 8 and 19 in MENX-associated rat pheochromocytoma. Int J Cancer.

[14] Stambolic V, Tsao MS, Macpherson D, Suzuki A, Chapman WB, Mak TW (2000). High incidence of breast and endometrial neoplasia resembling human Cowden syndrome in pten+/- mice. Cancer Res.

[15] Sweetser DA, Froelick GJ, Matsumoto AM (1999). Ganglioneuromas and renal anomalies are induced by activated RET(MEN2B) in transgenic mice. Oncogene.

[16] Yamasaki L, Bronson R, Williams BO, Dyson NJ, Harlow E, Jacks T (1998). Loss of E2F-1 reduces tumorigenesis and extends the lifespan of Rb1(+/-)mice. Nat Genet.

[17] Eisenhofer G, Bornstein SR, Brouwers FM (2004). Malignant pheochromocytoma: current status and initiatives for future progress. Endocr Relat Cancer.

[18] Dahia PL (2000). PTEN, a unique tumor suppressor gene. Endocr Relat Cancer.

[19] Di Cristofano A, Pandolfi PP (2000). The multiple roles of PTEN in tumor suppression. Cell.

[20] van Nederveen FH, Perren A, Dannenberg H (2006). PTEN gene loss, but not mutation, in benign and malignant phaeochromocytomas. J Pathol.

[21] Di Cristofano A, De Acetis M, Koff A, Cordon-Cardo C, Pandolfi PP (2001). Pten and p27KIP1 cooperate in prostate cancer tumor suppression in the mouse. Nat Genet.

[22] You MJ, Castrillon DH, Bastian BC (2002). Genetic analysis of Pten and Ink4a/Arf interactions in the suppression of tumorigenesis in mice. Proc Natl Acad Sci U S A.

[23] Sharpless NE, Bardeesy N, Lee KH (2001). Loss of p16Ink4a with retention of p19Arf predisposes mice to tumorigenesis. Nature.

[24] Cascon A, Ruiz-Llorente S, Fraga MF (2004). Genetic and epigenetic profile of sporadic pheochromocytomas. J Med Genet.

[25] Dannenberg H, Speel EJ, Zhao J (2000). Losses of chromosomes 1p and 3q are early genetic events in the development of sporadic pheochromocytomas. Am J Pathol.

[26] Edstrom E, Nord B, Carling T (2002). Loss of heterozygosity on the short arm of chromosome 1 in pheochromocytoma and abdominal paraganglioma. World J Surg.

[27] van Nederveen F, Korpershoek E, Deleeuw R (2009). Array-CGH in sporadic benign pheochromocytomas. Endocr Relat Cancer.

[28] Knudson AG (1993). Antioncogenes and human cancer. Proc Natl Acad Sci U S A.

[29] Salgia R, Skarin AT (1998). Molecular abnormalities in lung cancer. J Clin Oncol.

[30] Burkhart DL, Sage J (2008). Cellular mechanisms of tumour suppression by the retinoblastoma gene. Nat Rev Cancer.

[31] Tonks ID, Mould AW, Schroder WA (2010). Dual loss of rb1 and Trp53 in the adrenal medulla leads to spontaneous pheochromocytoma. Neoplasia.

[32] Hansen K, Farkas T, Lukas J, Holm K, Ronnstrand L, Bartek J (2001). Phosphorylation-dependent and -independent functions of p130 cooperate to evoke a sustained G1 block. EMBO J.

[33] Zamanian M, La Thangue NB (1993). Transcriptional repression by the Rb-related protein p107. Mol Biol Cell.

[34] Dyson N (1998). The regulation of E2F by pRB-family proteins. Genes Dev.

[35] Wakai J, Kizaki K, Yamaguchi-Yamada M, Yamamoto Y (2010). Differences in tyrosine hydroxylase expression after short-term hypoxia, hypercapnia or hypercapnic hypoxia in rat carotid body. Respir Physiol Neurobiol.

[36] Besson A, Dowdy SF, Roberts JM (2008). CDK inhibitors: cell cycle regulators and beyond. Dev Cell.

[37] Sherr CJ, Roberts JM (1999). CDK inhibitors: positive and negative regulators of G1-phase progression. Genes Dev.

[38] Solomon DA, Kim JS, Jenkins S (2008). Identification of p18 INK4c as a tumor suppressor gene in glioblastoma multiforme. Cancer Res.

[39] van Veelen W, Klompmaker R, Gloerich M (2009). P18 is a tumor suppressor gene involved in human medullary thyroid carcinoma and pheochromocytoma development. Int J Cancer.

[40] Franklin DS, Godfrey VL, Lee H (1998). CDK inhibitors p18(INK4c) and p27(Kip1) mediate two separate pathways to collaboratively suppress pituitary tumorigenesis. Genes Dev.

[41] Sandgren J, Diaz de Stahl T, Andersson R (2010). Recurrent genomic alterations in benign and malignant pheochromocytomas and paragangliomas revealed by whole-genome array comparative genomic hybridization analysis. Endocr Relat Cancer.

[42] Gratias S, Rieder H, Ullmann R (2007). Allelic loss in a minimal region on chromosome 16q24 is associated with vitreous seeding of retinoblastoma. Cancer Res.

[43] Kitamura Y, Shimizu K, Tanaka S, Ito K, Emi M (2000). Association of allelic loss on 1q, 4p, 7q, 9p, 9q, and 16q with postoperative death in papillary thyroid carcinoma. Clin Cancer Res.

[44] Kitamura Y, Shimizu K, Tanaka S, Ito K, Emi M (2000). Allelotyping of anaplastic thyroid carcinoma: frequent allelic losses on 1q, 9p, 11, 17, 19p, and 22q. Genes Chromosomes Cancer.

[45] Molatore S, Liyanarachchi S, Irmler M (2010). Pheochromocytoma in rats with multiple endocrine neoplasia (MENX) shares gene expression patterns with human pheochromocytoma. Proc Natl Acad Sci U S A.

[46] Powers JF, Evinger MJ, Zhi J, Picard KL, Tischler AS (2007). Pheochromocytomas in Nf1 knockout mice express a neural progenitor gene expression profile. Neuroscience.

[47] Marinoni I, Pellegata NS (2011). p27kip1: a new multiple endocrine neoplasia gene?. Neuroendocrinology.

[48] Georgitsi M (2010). MEN-4 and other multiple endocrine neoplasias due to cyclin-dependent kinase inhibitors (p27(Kip1) and p18(INK4C)) mutations. Best Pract Res Clin Endocrinol Metab.

[49] Airaksinen MS, The SM (2002). The GDNF family: signalling, biological functions and therapeutic value. Nat Rev Neurosci.

[50] Thakker RV (2001). Multiple endocrine neoplasia. Horm Res.

[51] Nikiforov YE (2008). Thyroid carcinoma: molecular pathways and therapeutic targets. Mod Pathol.

[52] Le LQ, Parada LF (2007). Tumor microenvironment and neurofibromatosis type I: connecting the GAPs. Oncogene.

[53] Williams VC, Lucas J, Babcock MA, Gutmann DH, Korf B, Maria BL (2009). Neurofibromatosis type 1 revisited. Pediatrics.

[54] Jacks T, Shih TS, Schmitt EM, Bronson RT, Bernards A, Weinberg RA (1994). Tumour predisposition in mice heterozygous for a targeted mutation in Nf1. Nat Genet.

[55] Tischler AS, Shih TS, Williams BO, Jacks T (1995). Characterization of Pheochromocytomas in a Mouse Strain with a Targeted Disruptive Mutation of the Neurofibromatosis Gene Nf1. Endocr Pathol.

[56] Bayley JP, van Minderhout I, Hogendoorn PC (2009). Sdhd and SDHD/H19 knockout mice do not develop paraganglioma or pheochromocytoma. PLoS One.

[57] Goncalves S, Paupe V, Dassa EP (2010). Rapid determination of tricarboxylic acid cycle enzyme activities in biological samples. BMC Biochem.

[58] Pellegata NS, Quintanilla-Martinez L, Keller G (2007). Human pheochromocytomas show reduced p27Kip1 expression that is not associated with somatic gene mutations and rarely with deletions. Virchows Arch.

[59] Joshi PP, Kulkarni MV, Yu BK (2007). Simultaneous downregulation of CDK inhibitors p18(Ink4c) and p27(Kip1) is required for MEN2A-RET-mediated mitogenesis. Oncogene.

[60] Kulkarni MV, Franklin DS (2011). N-Myc is a downstream target of RET signaling and is required for transcriptional regulation of p18(Ink4c) by the transforming mutant RET(C634R). Mol Oncol.

[61] Qin Y, Yao L, King EE (2010). Germline mutations in TMEM127 confer susceptibility to pheochromocytoma. Nat Genet.

[62] Comino-Mendez I, Gracia-Aznarez FJ, Schiavi F (2011). Exome sequencing identifies MAX mutations as a cause of hereditary pheochromocytoma. Nat Genet.

[63] Powers JF, Evinger MJ, Tsokas P (2000). Pheochromocytoma cell lines from heterozygous neurofibromatosis knockout mice. Cell Tissue Res.

[64] Powers JF, Schelling K, Brachold JM (2002). High-level expression of receptor tyrosine kinase Ret and responsiveness to Ret-activating ligands in pheochromocytoma cell lines from neurofibromatosis knockout mice. Mol Cell Neurosci.

[65] Powers JF, Schelling KH, Brachold JM, Tischler AS (2002). Plasticity of pheochromocytoma cell lines from neurofibromatosis knockout mice. Ann N Y Acad Sci.

[66] Pachnis V, Mankoo B, Constantini F (1993). Expression of the C-Ret Protooncogene during Mouse Embryogenesis. Development.

[67] Ohta S, Lai EW, Morris JC (2008). Metastasis-associated gene expression profile of liver and subcutaneous lesions derived from mouse pheochromocytoma cells. Mol Carcinog.

[68] Martiniova L, Kotys MS, Thomasson D (2009). Noninvasive monitoring of a murine model of metastatic pheochromocytoma: a comparison of contrast-enhanced microCT and nonenhanced MRI. J Magn Reson Imaging.

[69] Martiniova L, Ohta S, Guion P (2006). Anatomical and functional imaging of tumors in animal models: focus on pheochromocytoma. Ann N Y Acad Sci.

[70] Martiniova L, Schimel D, Lai EW, Limpuangthip A, Kvetnansky R, Pacak K (2010). In vivo micro-CT imaging of liver lesions in small animal models. Methods.

[71] Dhawan V, Ishikawa T, Patlak C (1996). Combined FDOPA and 3OMFD PET studies in Parkinson’s disease. J Nucl Med.

[72] Goldstein DS, Holmes C, Stuhlmuller JE, Lenders JW, Kopin IJ (1997). 6-[18F]fluorodopamine positron emission tomographic scanning in the assessment of cardiac sympathoneural function–studies in normal humans. Clin Auton Res.

[73] Pacak K, Eisenhofer G, Carrasquillo JA, Chen CC, Li ST, Goldstein DS (2001). 6-[18F]fluorodopamine positron emission tomographic (PET) scanning for diagnostic localization of pheochromocytoma. Hypertension.

[74] Pacak K, Eisenhofer G, Goldstein DS (2004). Functional imaging of endocrine tumors: role of positron emission tomography. Endocr Rev.

[75] Hoegerle S, Altehoefer C, Ghanem N (2001). Whole-body 18F dopa PET for detection of gastrointestinal carcinoid tumors. Radiology.

[76] Martiniova L, Cleary S, Lai EW, et al. Usefulness of [(18)F]-DA and [(18)F]-DOPA for PET imaging in a mouse model of pheochromocytoma.*Nucl Med Biol* 2011. 10.1016/j.nucmedbio.2011.07.007 PMC4713029 21958851

[77] Timmers HJ, Chen CC, Carrasquillo JA (2009). Comparison of 18 F-fluoro-L-DOPA, 18 F-fluoro-deoxyglucose, and 18 F-fluorodopamine PET and 123I-MIBG scintigraphy in the localization of pheochromocytoma and paraganglioma. J Clin Endocrinol Metab.

[78] Timmers HJ, Hadi M, Carrasquillo JA (2007). The effects of carbidopa on uptake of 6-18 F-fluoro-l-DOPA in PET of pheochromocytoma and extraadrenal abdominal paraganglioma. J Nucl Med.

[79] Martiniova L, Lai EW, Elkahloun AG (2009). Characterization of an animal model of aggressive metastatic pheochromocytoma linked to a specific gene signature. Clin Exp Metastasis.

[80] Brouwers FM, Elkahloun AG, Munson PJ (2006). Gene expression profiling of benign and malignant pheochromocytoma. Ann N Y Acad Sci.

[81] Kawakami K, Kawakami M, Husain SR, Puri RK (2002). Targeting interleukin-4 receptors for effective pancreatic cancer therapy. Cancer Res.

[82] Kunwar S, Prados MD, Chang SM (2007). Direct intracerebral delivery of cintredekin besudotox (IL13-PE38QQR) in recurrent malignant glioma: a report by the Cintredekin Besudotox Intraparenchymal Study Group. J Clin Oncol.

[83] Lai EW, Joshi BH, Martiniova L (2009). Overexpression of interleukin-13 receptor-alpha2 in neuroendocrine malignant pheochromocytoma: a novel target for receptor directed anti-cancer therapy. J Clin Endocrinol Metab.

[84] Averbuch SD, Steakley CS, Young RC (1988). Malignant pheochromocytoma: effective treatment with a combination of cyclophosphamide, vincristine, and dacarbazine. Ann Intern Med.

[85] Koch CA, Huang SC, Moley JF (2001). Allelic imbalance of the mutant and wild-type RET allele in MEN 2A-associated medullary thyroid carcinoma. Oncogene.

[86] Loh KC, Fitzgerald PA, Matthay KK, Yeo PP, Price DC (1997). The treatment of malignant pheochromocytoma with iodine-131 metaiodobenzylguanidine (131I-MIBG): a comprehensive review of 116 reported patients. J Endocrinol Invest.

[87] Munver R, Del Pizzo JJ, Sosa RE (2003). Adrenal-preserving minimally invasive surgery: the role of laparoscopic partial adrenalectomy, cryosurgery, and radiofrequency ablation of the adrenal gland. Curr Urol Rep.

[88] Sisson JC, Shapiro B, Meyers L (1987). Metaiodobenzylguanidine to map scintigraphically the adrenergic nervous system in man. J Nucl Med.

[89] Sisson JC, Shapiro B, Shulkin BL, Urba S, Zempel S, Spaulding S (1999). Treatment of malignant pheochromocytomas with 131-I metaiodobenzylguanidine and chemotherapy. Am J Clin Oncol.

[90] Sisson JC, Wieland DM (1986). Radiolabeled meta-iodobenzylguanidine: pharmacology and clinical studies. Am J Physiol Imaging.

[91] Glowniak JV, Kilty JE, Amara SG, Hoffman BJ, Turner FE (1993). Evaluation of metaiodobenzylguanidine uptake by the norepinephrine, dopamine and serotonin transporters. J Nucl Med.

[92] Martiniova L, Perera SM, Brouwers FM (2011). Increased uptake of [(1)(2)(3)I]meta-iodobenzylguanidine, [(1)F]fluorodopamine, and [(3)H]norepinephrine in mouse pheochromocytoma cells and tumors after treatment with the histone deacetylase inhibitors. Endocr Relat Cancer.

[93] Lu J, Kovach JS, Johnson F (2009). Inhibition of serine/threonine phosphatase PP2A enhances cancer chemotherapy by blocking DNA damage induced defense mechanisms. Proc Natl Acad Sci U S A.

[94] Martiniova L, Lu J, Chiang J (2011). Pharmacologic modulation of serine/threonine phosphorylation highly sensitizes PHEO in a MPC cell and mouse model to conventional chemotherapy. PLoS One.

[95] Bayley JP, Devilee P (2010). Warburg tumours and the mechanisms of mitochondrial tumour suppressor genes. Barking up the right tree?. Curr Opin Genet Dev.

[96] Bachireddy P, Bendapudi PK, Felsher DW (2005). Getting at MYC through RAS. Clin Cancer Res.

[97] Courtois-Cox S, Jones SL, Cichowski K (2008). Many roads lead to oncogene-induced senescence. Oncogene.

[98] Dahia PL, Ross KN, Wright ME (2005). A HIF1alpha regulatory loop links hypoxia and mitochondrial signals in pheochromocytomas. PLoS Genet.

[99] Lutz W, Leon J, Eilers M (2002). Contributions of Myc to tumorigenesis. Biochim Biophys Acta.

[100] Schlisio S, Kenchappa RS, Vredeveld LC (2008). The kinesin KIF1Bbeta acts downstream from EglN3 to induce apoptosis and is a potential 1p36 tumor suppressor. Genes Dev.

[101] Senderowicz AM, Sausville EA (2000). Preclinical and clinical development of cyclin-dependent kinase modulators. J Natl Cancer Inst.

